# Hypothesis: gonadal temperature influences sex-specific imprinting

**DOI:** 10.3389/fgene.2014.00294

**Published:** 2014-08-25

**Authors:** Paolo Prontera, Emilio Donti

**Affiliations:** Medical Genetics Unit, Department of Surgical and Biomedical Sciences, University of PerugiaPerugia, Italy

**Keywords:** genomic imprinting, hypothesis, temperature, gonads, RNA interference, evolution

## Abstract

Various explanations have been advanced for the evolution of genomic imprinting, the most popular of these being the parental conflict hypothesis. However, while this theory may explain why there has been selection for imprinting certain genes, it does not explain how the maternal and paternal genomes can be distinguished from each other. Here, we hypothesize that the temperature at which male and female gonads are physiologically exposed could be, at least for some loci, the primary factor leading to the different imprinting between the sexes.

## INTRODUCTION

In humans and many other mammals the expression of about 100 genes depends on their parent of origin. Specifically, when an allele is inherited from the father, its pattern of expression is different from when the same allele is inherited from the mother. This parent-of-origin-specific gene expression is known as genomic imprinting, a reversible epigenetic process driven by differential methylations of specific short DNA sequences (differentially methylated regions, DMRs; Ferguson-Smith, 2011; Smith and Meissner, 2013). DMRs can be divided into two classes: somatic or secondary DMRs acquire their methylated status after fertilization, whereas germline DMRs become differentially methylated during germ cell differentiation. Moreover, somatic DMRs may be tissue-specific and dependent on the presence of a germline DMR (Edwards and Ferguson-Smith, 2007).

Genomic imprinting is generally believed to be conserved in all mammals except for egg-laying monotremes, suggesting that it is closely related to placenta and fetal growth (Edwards and Ferguson-Smith, 2007; Ferguson-Smith, 2011; Smith and Meissner, 2013). Various explanations have been advanced for the evolution of imprinting, the most accepted being the parental conflict hypothesis (Moore and Haig, 1991), which suggests a strict correlation among genomic imprinting, viviparity, and placentation, due to competition between the parental genomes: the paternal genome strongly promotes fetal growth, whereas the maternal one limits the access to maternal resources.

While the parental conflict hypothesis may explain why there has been selection for imprinting certain genes, it fails to explain how the maternal and paternal genomes are distinguished.

## HYPOTHESIS AND DISCUSSION

Here, we hypothesize that, at least for some loci, the sex-specific epigenetic remodeling process might be influenced by the temperatures at which male and female gonads are exposed during the acquisition of a new imprinting.

This hypothesis is supported by three lines of evidence:

### IMPRINTING IS RESTRICTED TO ANIMALS HAVING MALE AND FEMALE GONADS AT DIFFERENT TEMPERATURES

Thermoregulation is a complex process that animals have developed in different ways. On the basis of the ability to control body temperature, animals can be divided into either homeothermic, heterothermic or poikilothermic. Homeotherms control their body temperature by varying their metabolic rates (endotherm), heterotherms exhibit characteristics of both homeothermy and poikilothermy, while poikilotherms are not able to control their body temperatures, which are mostly influenced by external temperatures (ectotherm; Grigg et al., 2004; Geiser, 2008). Recent studies have shown that endothermic thermoregulation evolved during the evolution of mammals and birds. Being so, mammals and birds are mainly homeotherms, while reptiles and fish are poikilotherms. There are few exceptions to this rule. For example, monotremes and bats are considered heterotherms (Grigg et al., 2004; Geiser, 2008). The control of body temperature has permitted these animals not only to be less subject to the influence of external temperature, but also to create and maintain different “temperature niches” inside their organisms, probably because this is evolutionarily advantageous.

This is the case of the mammal’s male gonads, which have a temperature 2–7°C lower than that of the average temperature of the body, unlike female gonads which are exposed to the same temperature of the body (Kleisner et al., 2010). This is the rule not only for a large group of mammals, including humans, endowed with the scrotum, but also for other non-scrotal mammals (about 1500 species, e.g., sloths, seals, dolphins, and whales) which have testes in a cooler position or which have a specialized cooling system (Werdelin and Nilsonne, 1999; Bininda-Emonds et al., 2007; Kleisner et al., 2010). For instance, whales and dolphins have internal testes that are kept cool due to the fact that the arteries going to the testes are near veins, bringing cooled venous blood from the skin (Rommel et al., 1992; Bedford, 2008), while bats have scrotal pouches that provide a temperature below that of the body (Jolly and Blackshaw, 1988). Therefore, the low temperature at which the male gonads are exposed is generally a common feature in mammals. It also plays a key role in spermatogenesis (Werdelin and Nilsonne, 1999; Bininda-Emonds et al., 2007; Kleisner et al., 2010), indeed men with undescended testes are infertile (Ivell, 2007). For the very few testicondy mammals (without scrota and having testes within the abdomen, e.g., elephants and hyraxes) we lack data on their gonadal temperature. However, since they have an average body temperature of 36.5°C, they are thought to have a special cooling system that allows for spermatogenesis (Gaeth et al., 1999).

Monotremes are also testicondy but differ from other testicondy mammals anatomically because their reproductive, urinary and digestive systems discharge into a common orifice, the cloaca. As well they differ physiologically because they have lower body temperatures on average (32°C; Moyal, 2001; Holland and Jackson, 2002; Kleisner et al., 2010). This lower body temperature allows for spermatogenesis without the aid of a special cooling system (Moyal, 2001; Holland and Jackson, 2002; Kleisner et al., 2010). Thus, in monotremes, there are no evident differences between the temperatures at which male and female gonads are exposed. Therefore, among mammals, it seems that only those with male and female gonads placed at different temperatures have genomic imprinting.

### IMPRINTING TAKES PLACE IN THE MALE AND FEMALE GONADS ONLY WHEN THEY HAVE REACHED DIFFERENT POSITIONS WITHIN THE BODY AND ONLY ONCE THEY HAVE ACQUIRED THEIR ANATOMICAL DIFFERENCES

The precise timings of imprint erasure and re-establishment in germ cells remains to be determined for many genes of different species and orders. To date, most studies have been carried out on *Mus Musculus*, so that most information has come from the analysis of this specie. As a whole, parental imprinting marks, transmitted by sperm and oocytes, are protected from the genome-wide reprogramming of the zygote. During the development of fetal gonads, a rapid DNA demethylation – process usually completed by mid-gestation – erases the parental imprinting in preparation of sex-specific *de novo* methylation (Ueda et al., 2000; Morgan et al., 2005; Ferguson-Smith, 2011; Smith and Meissner, 2013). But this process is quite different for maternal and paternal imprints. In fact, while the maternal methylation imprints are completely erased in male and female Primordial Germ Cells (PGCs), the paternal methylation imprint of *H19* appears to be preserved in a proportion of PGCs. In particular, *H19* displays biased biallelic expression in 11.5-dpc PGCs, with the more active allele being the maternal one (Szabo and Mann, 1995). It has also been reported that *H19* is partially methylated in embryonic germ (EG) cells derived from 11.5- and 12.5-dpc PGCs (Tada et al., 1998). This finding strongly supports the idea that at least some PGCs maintain the paternal *H19* imprinting, suggesting that the pre-existing methylation imprint is not completely erased. Also, whether other paternally methylated genes are partially methylated in PGCs remains to be determined (Ueda et al., 2000).

As for the timing of imprint erasure, also the timing for *de novo* methylation differs between sexes. In fact, it is commonly accepted that the establishment of maternally methylated DMRs occurs after birth in females, simultaneously, in the oocytes at the growing oocyte stage (Lucifero et al., 2004; Morgan et al., 2005; Ferguson-Smith, 2011; Smith and Meissner, 2013). In males there is evidence that *de novo* methylation starts late in fetal life or during the newborn period, and at least for some loci and some species, it seems to be acquired during post-natal life (Davis et al., 1999; Kerjean et al., 2000; Lucifero et al., 2004; Morgan et al., 2005; Boyano et al., 2008; Smith and Meissner, 2013; Suzuki et al., 2013). Boyano et al. (2008) have observed that genomic imprinting of *H19* genes is established at different stages of germ cells differentiation during post-natal life in male mice. Likewise, Suzuki et al. (2013) have reported that the *H19* DMR undergoes *de novo* methylation only after 34 days post-partum in the germ line of the marsupial *Tammar Wallaby*. Therefore, unlike the maternal imprints, it seems that paternally methylated *H19* (and maybe other paternally imprinted loci) partially retains imprints in PGCs till late developmental fetal stage and undergoes a complete re-methylation during this period, a process that continues also during the perinatal and post-natal life. Whereas the same *H19* locus acquires a new un-methylated status in the female gonads of mice at 16.5-dpc (Ueda et al., 2000).

In order to verify our hypothesis we would need to determine if, during this critical period of erasure/*de novo* methylation, the female and male gonads are exposed to the same or different temperatures. But, as for the imprinting, also the precise timing of the lower temperature acquisition in the male gonads remains to be defined. In homeotherms, central thermoregulatory mechanisms are already differentiated in fetal life (Asakura, 2004). In fact, during intrauterine life, the fetus is warmed by its own metabolic processes and can change its temperature when under temperature-stress conditions (Power, 1989; Asakura, 2004; Tzschentke and Rumpf, 2011). The precise timing is not known, but evidence suggests that mammalian embryos have developed thermoregulatory mechanisms already from the last months of the fetal period (Power, 1989; Asakura, 2004; Tzschentke and Rumpf, 2011). Moreover, in a review by Hughes and Acerini (2008) they have explored the embryology of testis descent in different mammals (humans, dogs, cattle, deer, horses, pigs, rabbits, mice), reporting that the male gonads are in the abdominal or inguinoscrotal phases well before the birth, except for dogs and mouse, which have testes before birth in the transinguinal phase and the inguinoscrotal phase is complete soon after birth. However, when the *H19* in the female gonads undergoes demethylation (16.5 dpc), the male gonads are in the abdominal or inguinoscrotal phases, and, at this point, also in the male gonads the *H19* is partially demethylated. Davis et al. (1999) observed that the timing of remethylation is different between the paternal and maternal alleles: while the paternal allele becomes hypermethylated during fetal stages, methylation of the maternal allele begins during perinatal stages and continues postnatally through the onset of meiosis. The Authors went on to suggest that in the absence of DNA methylation, other epigenetic mechanism(s) maintain parental identity at the *H19* locus during male germ cell development.

Here we hypothesize that this paternal “epi-methylation” structure is generated by the lower temperature at which the male gonads are exposed for a long period, before the fertilization (at least 16 years). If so, this temperature induced epi-methylation structure could be responsible for the early restoration of the methylated status in the paternal allele respect to the maternal one, which instead is exposed for a long time to a higher temperature and thus has a different epi-methylation profile. However, since the hypermethylation of the paternal allele appears during the last fetal period, we have to suppose that a slight temperature reduction occurs during this period. We have not found studies addressing the question of gonadal temperature in the fetus, but the thermoregulatory capability of the fetus, the different position of the testes compared to the ovaries, and the presence of the pampiniform plexus could generate a cooler environment (even of a few degrees: 0,5–1°C) with respect to body temperature. Indeed, the possibility of keeping the testes cool, even when they are located in the abdomen, is evident from cetaceans.

### TEMPERATURE CAN AFFECT CHROMATIN STRUCTURE AND GENE EXPRESSION

It has long been suspected that a link exists among temperature and RNA interference (RNAi), DNA methyltransferase (DNMT) activities and chromatin modification, all elements playing essential roles in epigenetic gene regulation (Fire, 1999; Kameda et al., 2004; Kloc et al., 2008; Campos et al., 2012; Oliver et al., 2012; Stępiński, 2012; Correia et al., 2013; Garolla et al., 2013).

RNA interference is thought to be an antiviral reaction, as well as an epigenetic regulator system involved in the suppression of internal transposons. In fact, plant, insect and mammalian cells have a temperature-sensitive RNAi, suggesting conservation throughout eukaryotic evolution (Fire, 1999; Kameda et al., 2004). Moreover, Kameda et al. (2004) have demonstrated that the hypothermic temperature-sensitive RNAi effect is a general feature of mammalian cells and the temperatures and degrees of sensitivity differ among target genes. Furthermore, they have suggested that replication-dependent and temperature-sensitive RNA-mediated silencing may contribute to environmental responses, as well as to the epigenetic inheritance of heterochromatin. Accordingly, Kloc et al. (2008) have reported that RNAi in fission yeast is inhibited at high temperatures, providing a plausible mechanism for epigenetic phenomena that seems to depend on replication and temperature, as in the vernalization of plants and PEV in animals. Kloc et al. (2008) have also suggested that RNAi is active in the S phase of the cell cycle, the latter which has major implications for epigenetic phenomena that depend on RNAi. These include transposon silencing, X inactivation and imprinting.

Regarding DNMTs, Campos et al. (2012) have reported that the expression levels of the *Dnmt3* genes in zebrafish (paralogs of the mammalian *DNMT3* genes) change when their embryos are incubated at different temperatures, thus suggesting a regulatory role for temperature also in *Dnmt3* expression and function.

Concerning the chromatin dynamics, a recent study analyzing the “sauna effect” on human spermatogenesis has shown that sperm chromatin is modified by high temperatures, even when briefly exposed (Garolla et al., 2013). This finding is in agreement with past studies on human fixed cells exposed to high temperatures, which observed temperature-induced chromatin changes (Cunningham et al., 1982; Jacobsen et al., 1988).

Collectively, all the main elements playing a role in the molecular pathway establishing genomic imprinting seem to be sensitive to temperature.

The most compelling precedent that highlights the role of temperature as a factor which can induce epigenetic modification specifically in germ cells, is the temperature-sensitive sex determination (TSD) present in some lizards, crocodiles and turtles. In these species, during the thermo-sensitive period, sex is susceptible to changes in specific temperature and, after this period, it cannot be reversed. The range of temperatures required to produce each sex is very narrow (Valenzuela, 2008). For example, in turtles having TSD, males are produced at lower incubation temperatures than females, the range being as narrow as 1–2°C. In lizards and crocodilians this pattern is reversed (Pen et al., 2010). Temperature-related gonadal differentiation is possible because specific genes in the germ line differentiation pathway displays temperature-influenced expressions (Valenzuela, 2008). To this regards, in a recent study on the turtle *Trachemys scripta*, Matsumoto et al. (2013) have evidenced that the female-producing temperature allows for demethylation at specific CpG sites of the promoter region of aromatase, leading to temperature-sensitive expression during gonadal development.

Overall, these studies demonstrate that minimal variations of temperature can induce different allele-specific epigenetic modifications, and that the gonads are particularly sensitive to this process. It is interesting to note that it has been recently reported that, in mouse blastocysts, heat stress caused an aberrant under-methylations in the paternally but not in the maternally imprinted *H19* and *Ifg2r* alleles (Zhu et al., 2008). This finding not only further highlights the relationship between temperature and epigenetic phenomena, but also suggests the presence of a specific parent-allele response to temperature variations, which is central to our hypothesis.

## FUTURE DIRECTIONS

In conclusion, we hypothesize that the acquisition of thermoregulation, the disappearance of the cloaca and the appearance of three distinct compartments (gastrointestinal, urinary and reproductive) in both eutherian and marsupial, are three main physiological developmental stages leading to differential gonadal temperatures between the sexes. Thus, these could be essential for the establishment and maintenance of genomic imprinting, at least for some specific loci.

In fact, there is growing evidence against the existence of a single signature for all DMRs (Abramowitz and Bartolomei, 2012). Being so, minimal temperature variation may act during a range of time only in some specific loci, as for example *H19*. In this locus the *de novo* imprinting seems to start (or restart) during spermatogonial differentiation in the late fetal period and be completed in the post-natal life (Davis et al., 1999; Kerjean et al., 2000; Boyano et al., 2008; Suzuki et al., 2013), when the male gonads, respectively, could be and are in hypothermia. Furthermore, in the same locus the parent specific methylation profile has been reported to be modified by temperature (Zhu et al., 2008).

Further studies are required to determine the pace in which male gonads in the fetus acquire hypothermia, and, at the same time, a broader study on the effects of temperature on RNAi, chromatin structure and dynamics, DNMT activities, and methylation establishment in germ cells, needs to be carried out to investigate for their possible dependences in establishing and maintaining genomic imprinting.

## AUTHOR CONTRIBUTIONS

Paolo Prontera developed the hypothesis, reviewed the literatures, wrote the manuscript; Emilio Donti supervised, discussed, and commented on the manuscript at all stage.

## Conflict of Interest Statement

The authors declare that the research was conducted in the absence of any commercial or financial relationships that could be construed as a potential conflict of interest.
